# Genome-Wide Identification and Expression Analysis of Members in the YT521-B Homology Domain-Containing RNA Binding Protein Family in *Ginkgo biloba*

**DOI:** 10.3390/plants13243589

**Published:** 2024-12-23

**Authors:** Han Wang, Jingjing Zhang, Sheng Yao, Xiang Cheng, Kongshu Ji, Qiong Yu

**Affiliations:** 1 State Key Laboratory of Tree Genetics and Breeding, Nanjing Forestry University, Nanjing 210037, Chinaksji@njfu.edu.cn (K.J.); 2 Key Open Laboratory of Forest Genetics and Gene Engineering of National Forestry & Grassland, Nanjing Forestry University, Nanjing 210037, China; 3 Co-Innovation Center for Sustainable Forestry in Southern China, Nanjing Forestry University, Nanjing 210037, China; 4 Beijing National Laboratory for Molecular Sciences, Beijing 100190, China

**Keywords:** *N^6^*-methyladenosine (m^6^A), *G. biloba*, YTH, readers

## Abstract

*N^6^*-methyladenosine (m^6^A) is a widespread post-transcriptional modification of RNA in eukaryotes. The conserved YTH-domain-containing RNA binding protein has been widely reported to serve as a typical m^6^A reader in various species. However, no studies have reported the m^6^A readers in *Ginkgo biloba* (*G. biloba*). In this study, a systematic analysis of the m^6^A reader (YTH) gene family was performed on *G. biloba*, identifying 10 *YTH* genes in its genome. Phylogenetic analysis of protein-coding sequences revealed that *YTH* genes from *G. biloba* could be classified into two subgroups: *GbDC1* and *GbDC2* in *GbDC* and *GbDF1-8* in *GbDF*, each with similar motifs and gene structures. In *G. biloba*, the predicated aromatic cage pocket of the YTH domains in the *YTH* gene family is uniformly composed of tryptophan residues (WWW). Subcellular localization experiments verified that *GbDC1* is indeed localized in the nucleus, while *GbDF1* is localized in both the nucleus and the cytoplasm. The expression patterns of the identified m^6^A reader genes showed a wide distribution but were tissue-specific. Most genes were highly expressed in leaves, followed by the stem, while the lowest expression tendency was found in the roots. Cis-regulatory element analysis predicted the possible functions of *YTH* genes in *G. biloba*, which were mainly responsive to plant hormones such as ABA and MeJA, as well as stress responses. Furthermore, the expression levels of *YTH* genes indeed changed significantly after ABA, MeJA, and NaCl treatments, suggesting that they can be affected by these abiotic factors. In addition, the PLAAC prediction results indicate that prion domains exist in *GbDF1*, *GbDF2*, *GbDF3*, *GbDF4*, *GbDF6*, *GbDF7*, *GbDF8*, and *GbDC1*, and phase separation is possible. This study provides a foundation for further investigation of the effects of m^6^A methylation on gene expression regulation in *G. biloba* and other forest trees.

## 1. Introduction

The regulation of gene expression is crucial for eukaryotes by affecting the growth, development, and other processes of organisms in varying degrees. Recently, chemical modifications in RNA as the rising epigenetic regulators have been increasingly reported to display an important impact on the regulation of gene expression. Among all RNA chemical modifications, m^6^A is the most common and widespread epigenetic modification in eukaryotic mRNAs, and it plays a vital role in many biological processes, including cell division in organ primordia, morphogenesis, flowering, as well as resistance to stresses in plants by disrupting the secondary structure of mRNA in the region where it is located and recognizably recruiting proteins such as YTH proteins to determine the fate of RNA [1]. What is more, in plants, m^6^A also influences their sex determination [2,3]. Currently, there are many studies proving the function of m^6^A, especially the m^6^A recognition proteins in plants, but there is still a need for further studies. Therefore, it is necessary to study plant YTH proteins to enrich our understanding of m^6^A regulatory functions in plant life.

The YTH (YT521-B homology) domain, characterized by 14 invariant residues within a helix/sheet structure, is related to the pseudouridine synthase and archaeosine transglycosylase (PUA) domain [4]. The conservation of aromatic residues in the β-sheets of the YTH domain is similar to that of the RNA recognition motif (RRM) domain [5]. In the RRM domain, conserved aromatic residues located in the β-sheet are crucial for RNA binding. These things considered, the YTH domain shows remarkable conservation across a wide species range, with 14 invariant and 19 highly conserved residues [5]. It has been shown in previous studies that YTH proteins, with the conversed YTH domain that has the ability to bind m^6^A, are important components of the m^6^A modification system. Furthermore, according to this study of the molecular recognition mechanism of m^6^A by YTH proteins, it was demonstrated that m^6^A modification sites varied among different tissues of plants. Due to the complexity of YTH proteins in plants, we hypothesized that YTH proteins in plants might bind to different m^6^A sites with selective preferences [6,7]. It has been found that YTH proteins in *Arabidopsis thaliana* and rice are required for organogenesis and flower timing as well as stress responses [8]. In tomatoes, the data suggest that YTH proteins result in the augmented production of aroma-associated volatiles [9] and have an important role in regulating tomato leaf senescence [10]. Also, YTH proteins are associated with powdery mildew resistance in apples [11]. As plants contain more YTH proteins than animals, many of their biological functions are still unknown, even in model plants. Therefore, it is worth revealing the functions of these YTH proteins in plants, especially those of forest trees which confer larger scales of distribution across our planet and make a huge contribution to the health of ecology.

*G. biloba* is one of the oldest tree species in China with high ornamental, timber, and medical value. Since the Song Dynasty, *G. biloba* has been used as a medicine in China and, currently, it is widely applied in the clinical therapy of cardiovascular diseases with good efficacy [12], which is one of the most successful cases of botanical medicine developed by modern science and technology in the world over the past 50 years. Due to the limited genetic diversity and low distribution of wild populations of *G. biloba*, it has been classified as an endangered and nationally protected plant. Hence, increasing studies have been devoted to investigating the genetic and developmental science of *G. biloba* in order to promote the breeding and protection of *G. biloba.* However, no studies related to m^6^A, the vital plant epigenetic regulatory module, have been reported yet. In this study, we focused on the study of m^6^A from the perspective of YTH proteins, the m^6^A recognition protein, aiming to shed new light on the molecular science and breeding of *G. biloba*. Here, a total of 10 *G. biloba YTH* genes were identified and their molecular properties as well as expression patterns across different tissues and under abiotic stresses were analyzed in detail. According to our results, it is obvious that YTH proteins have various impacts on the development of and stress responses in *G. biloba*, providing a theoretical basis for further understanding m^6^A’s influence on *G. biloba*’s growth, development, and stress resistance.

## 2. Materials and Methods

### 2.1. Identification of the G. biloba YTH Gene Family

In order to identify the *G. biloba YTH* gene family from the *G. biloba* genome database, we performed BLASTP [13] and HMMER [14] searches, and we then screened and filtered the redundant sequences as well as sequences without complete reading frames. Finally, 10 active protein sequences were identified and analyzed for their physicochemical properties. The molecular masses and isoelectric points of the GbYTH proteins were predicted using the web tool ExPASy [15] (https://web.expasy.org/compute_pi/, accessed on 11 October 2024). In addition, the subcellular localization of the *YTH* genes was predicted using WoLF PSORT [16] (https://wolfpsort.hgc.jp/, accessed on 11 October 2024).

### 2.2. Phylogenetic Analysis and Chromosome Localization

A maximum likelihood phylogenetic tree was constructed with 1000 bootstrap replications using MEGAX [17] (https://www.megasoftware.net/, accessed on 14 October 2024) and visualized by FigTree (http://tree.bio.ed.ac.uk/software/, accessed on 14 October 2024). The positional information of *GbYTHs* on chromosomes was extracted from the annotation files obtained from the *G. biloba* database, and the length of each chromosome was obtained from the NCBI website, and their distribution on chromosomes was obtained by mapping using MapChart [18] software (Version 2.32).

### 2.3. Gene Structure, Conserved Motif Analysis, and Multiple Sequence Alignments

The conserved motifs of *GbYTHs* were analyzed through the MEME online website (https://meme-suite.org/meme/doc/meme.html, accessed on 15 October 2024) [19], with a maximum motif retrieval value of 10 and default settings for other parameters. The motifs were visualized in TBtools (Version 2.142) [20] using the gff file of *GbYTH* genes with the MEME online prediction file mast.xml. Multiple sequence alignment of the obtained protein sequences of *G. biloba YTH* family was performed using ClustalW (https://www.genome.jp/tools-bin/clustalw, accessed on 15 October 2024), and the results were then visualized by using the ESPript 3.0 (https://espript.ibcp.fr/ESPript/cgi-bin/ESPript.cgi, accessed on 15 October 2024) online website.

### 2.4. Liquid–Liquid Phase Separation Prediction

Predictions of prion structural regions in the protein sequences of the *G. biloba YTH* gene family were made using the PLAAC online tool (PLAAC; http://plaac.wi.mit.edu/, accessed on 16 October 2024) [21], and the predictions were visualized using artificial intelligence.

### 2.5. Prediction of Cis-Regulatory Elements of the G. biloba YTH Genes

The 2000bp promoter sequences of the translation start site of the *G. biloba YTH* genes were extracted, and cis-acting elements were analyzed through the PlantCARE online website (https://bioinformatics.psb.ugent.be/webtools/plantcare/html/, accessed on 16 October 2024) to select cis-acting elements related to stress and hormone response. Their number was clustered and analyzed by using TBtools [20].

### 2.6. Subcellular Localization of GbDC1 Protein

The coding sequences of the *GbDC1* and *GbDF1* genes were amplified using the corresponding primers: GbDC1-F, GbDC1-R and GbECT2-F, GbECT2-R, respectively (Table S5). These amplified sequences were then inserted between the XbaI and SalI restriction sites of the 1305 vector, and eGFP was inserted at the C-terminus. Vectors carrying 35S::GbDC1-eGFP, 35S::GbECT2-eGFP, and 35S::eGFP were introduced into Agrobacterium cells GV3101. Healthy tobacco leaves (grown for 30 days) were infiltrated with Agrobacterium-mediated transient transformation [22]. Leaf samples (0.5 cm × 0.5 cm) were observed under a laser confocal microscope (LSM710, Zeiss, Jena, Germany).

### 2.7. Plant Materials and Treatments

Expression of *YTH* genes was analyzed in roots, stems, and leaves of six-month-old ginkgo seedlings cultivated in the Key Laboratory of Forest Tree Genetic Breeding, Nanjing Forestry University (32°04′43.05″ N, 118°49′1.70″ E). After spraying ABA and MeJA at concentrations of 100 μM and 100 μM [23], respectively, and salinity treatment with 100 mM NaCl for 6 h [24] on six-month-old *G. biloba* seedlings, leaf samples were collected at 0 h (untreated), 6 h, 12 h, 24 h, and 48 h and then immediately frozen in liquid nitrogen and stored at −80 °C. Three seedlings with uniform growth were selected as three biological replicates for each treatment.

### 2.8. RNA Extraction and RT-qPCR Analysis

RNA was extracted from *G. biloba* tissues using an RNA extraction kit (Vazyme, Nanjing, China). The extracted RNA was then analyzed by 1.2% agarose gel electrophoresis to confirm its quality and integrity. cDNA was diluted at a 1:20 concentration. Primers were designed using Primer 5 software (Version 5.0) based on the sequence of *G. biloba* in the CDS database. *GbGAPDH* was used as a reference control gene. Each PCR mixture (10 μL) consisted of 1 μL of diluted cDNA (20-fold dilution), 5 μL of SYBR Green Real-time PCR Master Mix (Yeasen, Shanghai, China), 0.4 μL of each primer (10 μM) (Table S6), and 3.2 μL of ddH_2_O. The RT-qPCR reactions were performed under the following conditions: 1 cycle at 98 °C for 3 min, followed by 40 cycles at 95 °C for 15 s, 60 °C for 30 s, and 72 °C for 30 s. Three biological and three technical replicates were analyzed for each sample. Finally, the relative expression of the genes was calculated using the 2^−ΔΔCt^ method [25], analyzed for significance, and plotted using GraphPad software (Version 9.5).

## 3. Results

### 3.1. Identification of the G. biloba YTH Gene Family

Through HMMER and BLASTP search, we finally identified 10 *YTH* genes from *G. biloba* genome (Table 1 and Table S1). The proteins encoded by this gene family ranged in length from 393 amino acids (*GbDC2*) to 779 amino acids (*GbDF6*), with molecular weights ranging from 44.173 kDa (*GbDC2*) to 85.087 kDa (*GbDF6*) and theoretical isoelectric points ranging from 5.05 (*GbDF8*) to 9.24 (*GbDC2*). Among these, *GbDF5* and *GbDC2* have isoelectric points greater than 7, while the remaining eight genes have isoelectric points of less than 7. This shows that the *G. biloba* YTH proteins span a wide range of amino acid lengths and molecular weights, and most of them are acidic proteins.

### 3.2. Phylogenetic Analysis of the G. biloba YTH Gene Family

**Figure 1:**
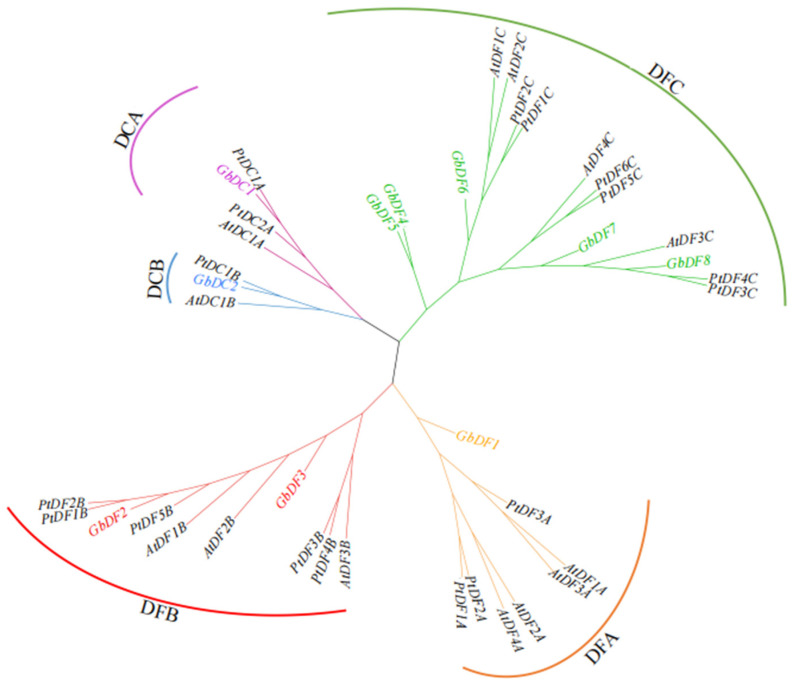
Phylogenetic relationships among different YTH-domain-containing proteins from various species, including *Ginkgo biloba*, *Arabidopsis thaliana*, and *Populus trichocarpa.* YTH family members were clustered into two groups and further divided into two and three subgroups distinguished by different colors, respectively.

We constructed a phylogenetic tree of the *G. biloba YTH* gene family using the sequences of 13 *Arabidopsis thaliana YTH* gene family proteins and 17 *Populus trichocarpa YTH* gene proteins (Figure 1 and Table S2). We categorized the *G. biloba YTH* gene family into DF and DC subgroups with reference to *Arabidopsis thaliana* [26] and previous studies of the *YTH* gene family. The results showed that *G. biloba* YTHDF proteins could be classified into three subgroups: DFA, DFB, and DFC, respectively. Among them, eight *G. biloba* YTH proteins belonged to the DF subgroup, one *G. biloba* YTH protein was located within the DCA subgroup and DCB subgroup, respectively. Furthermore, we found that there are some proximity groups between *G. biloba* and *Populus trichocarpa*, suggesting that they are straight homologous proteins, all of which exist in DC subgroups, such as *GbDC1-PtDC1A* and *GbDC2-PtDC1B*.

### 3.3. Localization of Chromosomes

**Figure 2:**
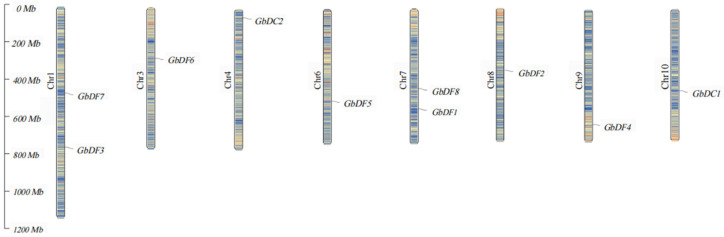
Distribution of *YTH* genes on *G. biloba* chromosomes. The size of a chromosome is indicated by its relative length. The scale on the left represents chromosome length in megabases (Mb).

Based on the genome annotation files of *GbYTHs* obtained in the Ginkgoaceae database, the physical localization of the *G. biloba YTH* gene family was organized and analyzed using TBtools software (Version 2.142). The results showed that the 10 members of the *YTH* gene family were distributed on eight chromosomes of *G. biloba*, with none present on chromosomes 02, 05, 11, and 12 (Figure 2). Specifically, *GbDF2*, *GbDF4*, *GbDF5*, *GbDF6*, *GbDC1*, and *GbDC2* were each localized to individual chromosomes, exhibiting an uneven and random distribution pattern.

### 3.4. Structural Characterization of the G. biloba YTH Gene Family

**Figure 3:**
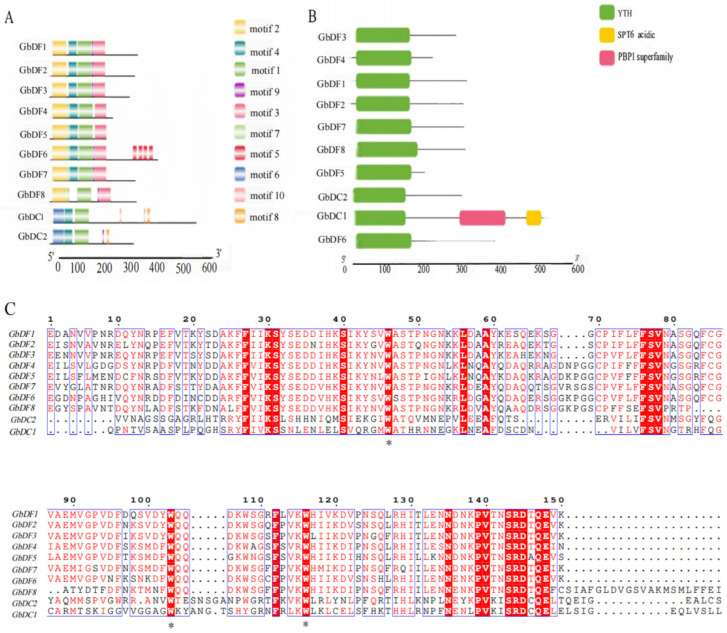
The domains, motif structures, and multiple sequence alignments of *G. bilaba* GbYTHs. (**A**) Conserved motifs in GbYTHs. Different motifs are distinguished by different colors. (**B**) Conserved domains of GbYTHs. Includes three different domains: YTH, SPT6 acidic, and PBP1 superfamily. YTH is a region that specifically binds to m^6^A-modified RNA. SPT6 is a transcriptional elongation factor involved in regulating RNA polymerase II activity, chromatin remodeling, and histone modification. PBP1 is a family of enzymes capable of peptidoglycan synthesis. (**C**) Multiple sequence alignments of YTH domain in *G. bilaba* YTH family proteins. Asterisks indicate the tryptophan position. The blue box indicates the conserved sequence, while the red highlight marks the identical sequence.

Conserved motif analysis showed that the 10 *G. biloba YTH* genes included 10 conserved motifs (Figure 3A and Table S3). Different subfamilies in the *GbYTH* gene family showed distinct patterns of gene structure. Members in each subgroup of the *GbYTH* gene family shared similar gene structures and lengths. Notably, all *G. biloba YTH* genes contained motif 1, and there were significant differences among members of different subfamilies. For instance, motif 2 and motif 3 were exclusively present in the DF family, while motif 6 and motif 8 were unique to the DC family. Furthermore, the sequences of motif 2 and motif 3 were longer than those of motif 6 and motif 8. Additionally, there were variations in the gene structures among members of the same subfamily. For example, within the DFC subfamily, *GbDF4*, *GbDF5*, *GbDF6*, and *GbDF8* contained motif 7, whereas *GbDF7* did not. Only the *GbDF8* gene lacked motif 4, while the remaining nine genes possessed motif 4 (Figure 3A).

Analysis of *G. biloba* YTH proteins showed that the YTH domain, responsible for binding to m^6^A, was the sole recognizable module in the sequences of the proteins, with the exception of GbDC1. Each GbYTH protein has a typical functional YTH domain. The GbDC1 protein sequence contained not only the YTH domain but also an acidic N-terminal region akin to that of the SPT6 protein and a PBPI superfamily domain (Figure 3B).

In addition, we performed multiple sequence alignment comparisons of 10 protein sequences from the *G. biloba* YTH proteins. The comparison results indicated that many functional sites of *G. biloba* YTH family proteins were conserved and had conserved aromatic cages. It is worth noting that the aromatic cages of the YTH domain in *G. biloba* YTH family are composed of tryptophan residues (WWW) (Figure 3C).

### 3.5. Cis-Regulatory Elements Analysis of the G. bilaba YTH Gene Promoters

**Figure 4:**
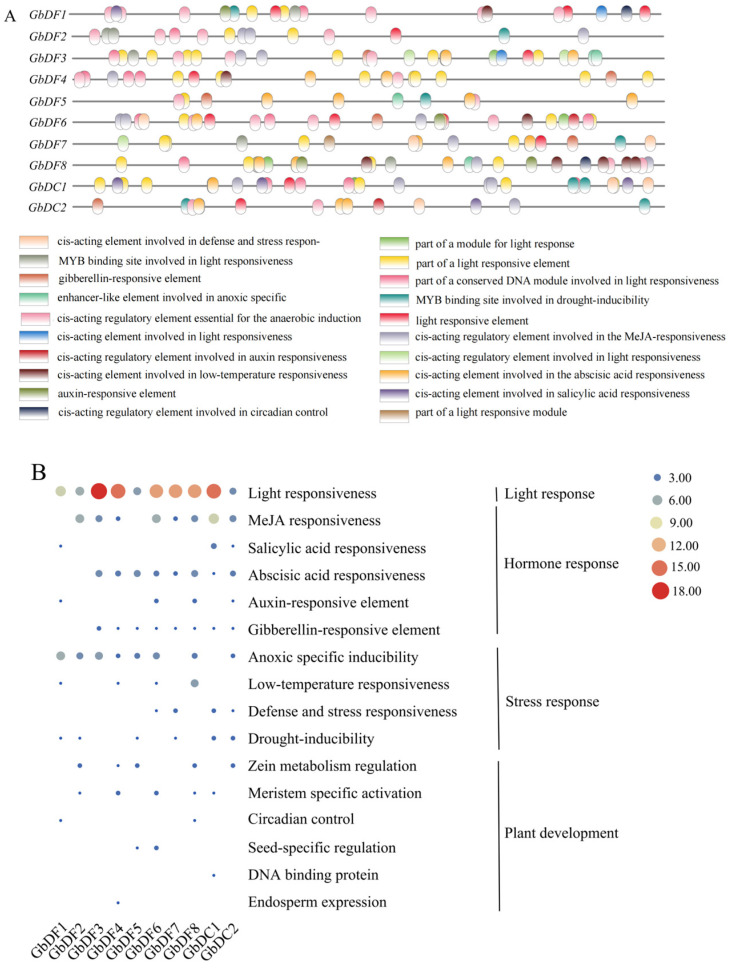
Promoter analysis of the *G. biloba YTH* family. (**A**) Putative cis-regulatory elements existed in the 2000 bp upstream region of *GbYTH* gene promoter. Each of the predicted cis-regulatory elements is represented by a different color. (**B**) Quantitative analysis of the number of light/stress modules present in the *GbDF* genes. Different colors and sizes represent different proportions.

To better understand the response of members of the *YTH* gene family to various abiotic stresses and to infer the functions of these YTH proteins in biological growth and development, the online tool PlantCARE was used to analyze the promoter cis-regulatory elements of the upstream 2.0 kb sequence of the translation initiation site of the *YTH* gene family. The results showed that various core cis-regulatory elements could be found in the promoter region of *YTH* genes (Figure 4 and Table S4). Notably, most of these cis-regulatory elements were involved in light responsiveness, plant growth hormones such as gibberellins, and abscisic acid and stress responses as well as anaerobic induction. These findings indicated that the *YTH* genes family may play important roles in regulating the growth and development of *G. biloba* through its involvement in light response, hormone response, and response to environmental stresses.

### 3.6. Liquid–Liquid Separation of YTH Gene Family Proteins

**Figure 5:**
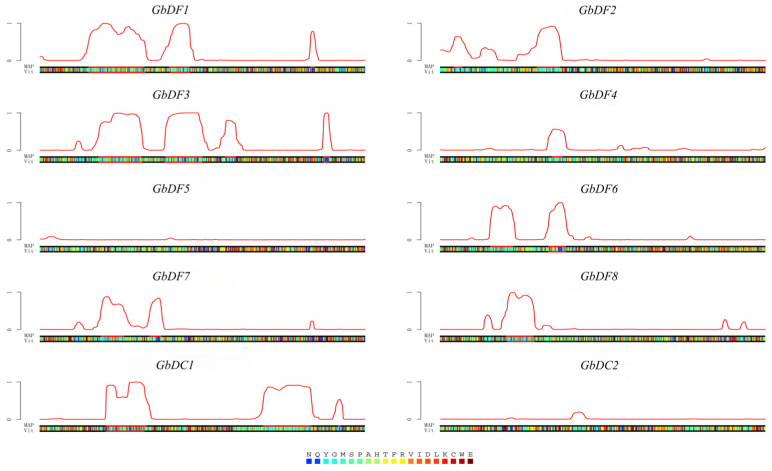
Disordered regions predicted with prion-like amino acid combinations. The red line indicates the prediction of the structure region of the prion. If the red line is located in the non-baseline region, it indicates that phase transition is very likely to occur.

Liquid–liquid phase separation (LLPS) is a phenomenon in which two solutions fail to dissolve into a homogeneous phase, resulting in phase separation, similar to oil floating on water in physics. In contrast to physics and chemistry, the occurrence of “liquid-liquid phase separation” in biological macromolecules has newly been discovered in recent years, resulting in the formation of a distinct working region and the exercise of specific biological function. Studies have demonstrated that YTHDF1 is able to degrade mRNA via LLPS [27] and that m^6^A enhances the phase separation of YTHDF proteins [28]. Therefore, in this study, we used the online tool PLAAC to predict whether the *G. biloba YTH* gene family protein sequence contains prion domains and, thus, analyze its phase transition potential. The results revealed the presence of one to four prion structural regions in the predicted sequences of GbDF1-4, GbDF6-8, and GbDC1 protein, respectively. This suggests a high probability that these genes undergo phase transition (Figure 5).

### 3.7. Subcellular Localization of GbDF1 and GbDC1 Protein

**Figure 6:**
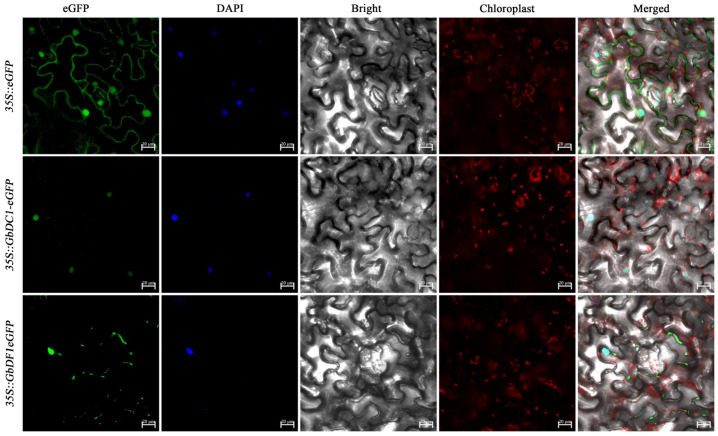
Analysis of subcellular localization of *GbDC1* and *GbDF1*. 35S::GbDC1-eGFP and 35S::GbECT2-eGFP were transiently expressed in tobacco leaves by transformed Agrobacterium tumefaciens injections. The green fluorescence of the eGFPs (first from the left), 4′,6-diamidino-2-phenylindole (DAPI) staining (second from the left), bright field (third from the right), chloroplast autofluorescence (second from the right), and merged field (first from the right) were observed using laser confocal microscopy. The scale bar is 20 μm.

To better understand the cellular functions of these GbYTH proteins, it is necessary to confirm their subcellular localizations. Hence, we selected *GbDF1* and *GbDC1*, homologs of *Arabidopsis thaliana*’s *ECT2* and *CPSF30-L*, as representative to perform subcellular localization experiments. Agrobacterium tumefaciens strains containing 35S::GbDC1-eGFP plasmid, 35S::GbDF1-eGFP plasmid, and 35S::eGFP empty vector were injected into healthy tobacco leaves, respectively (Figure 6). Fluorescence confocal microscopy revealed expression of 35S::eGFP empty vector in cell membranes, nuclei, and the cytoplasm. The 35S::GbDC1-eGFP plasmid produced green fluorescent signals exclusively in the nucleus of tobacco cells, indicating that the protein encoded by the *GbDC1* gene is mainly localized in the nucleus, similar to its homolog *CPSF30-L* in *Arabidopsis*. In contrast, the fluorescent signals derived from the 35S::GbDF1-eGFP plasmid were detected in both the nucleus and the cytoplasm of tobacco cells, suggesting that the *GbDF1* is expressed in both the nucleus and cytoplasm, consistent with ECT2 in *Arabidopsis*.

### 3.8. Expression of YTH Genes in Different Tissues of G. biloba

**Figure 7:**
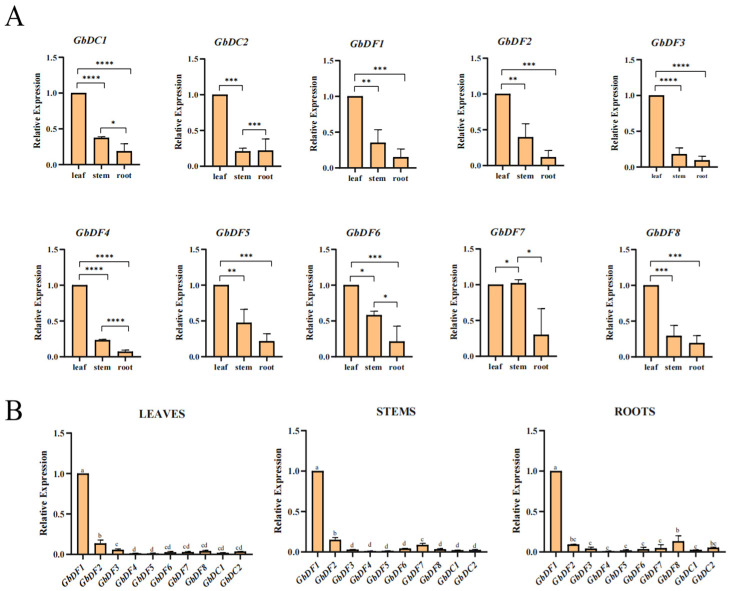
Tissue-specific expression patterns of 10 *G. biloba YTH* genes. Expression of *YTH* genes were analyzed in roots, stems, and leaves of six-month-old ginkgo seedlings. (**A**) Expression of *G. biloba YTH* genes in different tissues of *G. biloba*. The relative expression level was normalized against the expression level in leaf as the control. (**B**) Comparison of expression levels of 10 *G. biloba YTH* genes in leaf, stem, and root. The relative expression level was normalized against the expression level of *GbDF1*. Different numbers of asterisks (*) indicate significant differences (* *p* < 0.05, ** *p* < 0.01, *** *p* < 0.001, and **** *p* < 0.0001). Data are shown as mean ± SE, with three biological replicates. Different letters represent different levels of significance. Different letters represent significant differences between two groups, while the same letter represents no significant differences between the two groups.

To better reveal the physiological functions of *GbYTH* genes in *G. biloba*, we analyzed the expression of 10 *GbYTH* genes in the roots, stems, and leaves of *G. biloba*. The results showed that the expression of *GbYTH* genes varied across different tissues, but most of the genes showed similar expression patterns, with the highest expression in leaves, followed by stems, and the lowest expression in roots (Figure 7A). The expression of *GbDF4*, *GbDF5*, and *GbDC1* was low in all tissues, whereas *GbDF1* exhibited the highest expression patterns in all tissues, followed by *GbDF2*, which was expressed significantly higher in different tissues compared to other genes (Figure 7B).

### 3.9. Stress-Responsive Expression of the G. biloba YTH Genes

**Figure 8:**
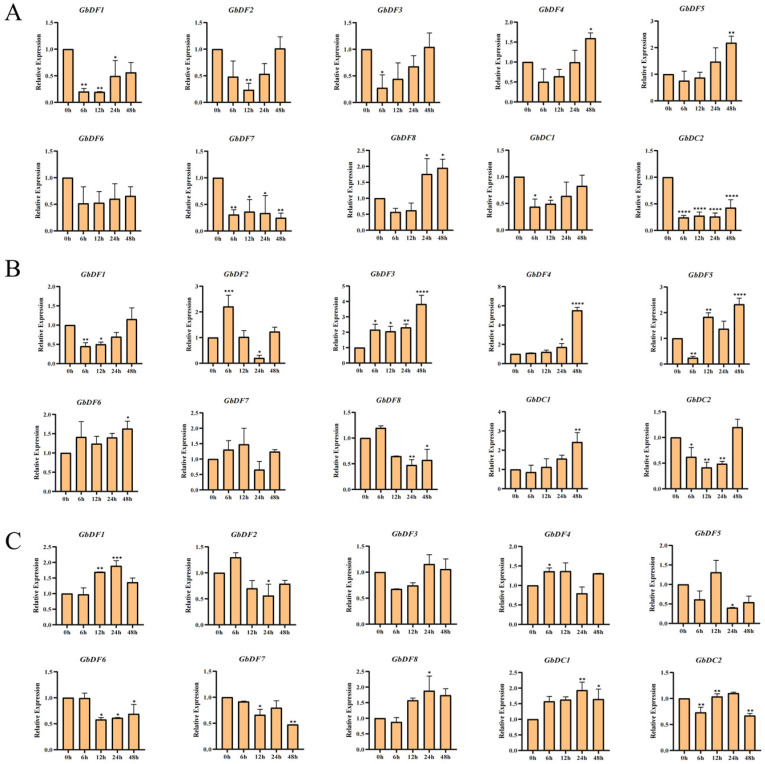
RT-qPCR analysis of *G. biloba YTH* gene expression under 100 μM ABA, 100 μM MeJA, and 100 mM NaCl treatments. Expression of *YTH* genes was analyzed in roots, stems, and leaves of six-month-old ginkgo seedlings. (**A**) The expression patterns of *G. biloba YTH* genes under ABA treatment. (**B**) The expression patterns of *G. biloba YTH* genes under MeJA treatment. (**C**) The expression patterns of *G. biloba YTH* genes under NaCl treatment. The relative expression level was measured with the expression level of 0 h of treatment as the control. Different numbers of asterisks (*) indicate significant differences (* *p* < 0.05, ** *p* < 0.01, *** *p* < 0.001, and **** *p* < 0.0001). Data are shown as mean ± SE, with three biological replicates.

By analyzing the cis-regulatory elements of the *G. biloba YTH* gene promoters, we found that the promoter region contains a significant number of plant hormone response elements, such as ABA and MeJA, as well as stress response cis-regulatory elements. Consequently, we subjected *G. biloba* to ABA, MeJA, and NaCl treatments to investigate the responses of the *YTH* genes to these stresses. After ABA treatment, the expressions of all genes except *GbDF6* were significantly changed and most genes showed a trend of decreasing at approximately 6 h and then increasing at 24 h. Notably, the expression of *GbDF7* dramatically decreased at 6 h without any increase (Figure 8A). After MeJA treatment, the expression of *GbYTH* genes showed more diverse patterns: *GbDC2*, *GbDF1*, and *GbDF5* showed a trend of decreasing and then increasing; *GbDF3*, *GbDF4*, *GbDF6*, and *GbDC1* exhibited a trend of continuous increasing; and *GbDF2*, *GbDF7*, and *GbDF8* showed a trend of increasing, then decreasing, and finally increasing (Figure 8B). After NaCl treatment, the expressions of all genes except *GbDF3* and *GbDF5* were significantly changed; the expression of *GbC1*, *GbDF1*, and *GbDF8* was elevated and the expression of *GbDF6* and *GbDF7* was decreased (Figure 8C). Obviously, most *GbYTH* genes have been proved to change due to the response to those abiotic stresses.

## 4. Discussion

The regulation of gene expression occurs at both transcriptional and post-transcriptional levels and affects various life processes in organisms. In recent years, studies on RNA epigenetics have demonstrated the important role of chemical modifications such as m^6^A, the first discovered RNA epigenetic mark, in gene expression regulation. RNA binding proteins containing the YTH domain play a crucial role in regulating the translation and stability of m^6^A-modified RNAs. With the development of plant RNA epigenetics, YTH proteins have been studied in various plants, including *Arabidopsis thaliana* [26], cucumber [29], tomato [30], common wheat [31], rice [32], etc. Nevertheless, to date, there have been no relevant reports on YTH proteins in *G. biloba*. Therefore, to better understand the role of m^6^A modification in *G. biloba*, the *CbYTH* genes were identified and analyzed using bioinformatics methods in this study.

A total of 10 *G. biloba YTH* gene family members were identified in this study and they were unevenly distributed on eight chromosomes. In mammals, YTH proteins are categorized as YTHDC1, YTHDC2, and YTHDF, with only one YTHDF subfamily homologous protein in most invertebrates and three YTHDF homologous proteins, YTHDF1, YTHDF2, and YTHDF3, in most vertebrates [33], which are strongly associated with cancers through their binding to the m^6^A site of RNA and affecting the RNA processing progress such as splicing, stability, or translation [34]. Compared to animals, the variation in YTH proteins in plants is more complex; apart from the absence of YTHDC2 homologous proteins in plants, YTHDC1 homologous proteins and YTHDF homologous proteins vary in homology and quantity across different plants. For example, most of dicotyledonous plants have more than two YTHDC1 proteins, whereas monocotyledonous plants only have one to two YTHDC1 proteins. The larger number of *YTH* genes identified in *G. biloba* is in line with previous findings that plants contain more *YTH* genes, such as the genomes of rice, tomato, and wheat harboring 12 [32], 9 [30], and 39 [31] YTH domain genes, respectively, potentially indicating more complex regulatory mechanisms or functional redundancy among m^6^A recognition proteins. Based on the structural similarity of the *YTH* gene family and the results of previous studies, we analyzed the evolutionary relationship between *G. biloba* and *A. thaliana*, as well as other species, and finally divided the *G. biloba YTH* gene family into DC subgroups and DF subgroups. The DC subgroups contain two subgroups, DCA and DCB, whereas the DF subgroup contains three subgroups, DFA, DFB, and DFC. Notably, the DF subgroup harbors more *YTH* genes, consistent with the findings in *A. thaliana* [26] and wheat [31], further suggesting significant variation in the homology and number of *YTH* family proteins. And our results show that the YTH proteins of *Populus trichocarpa* have higher homology with that of *G. biloba* than that of *Arabidopsis thaliana*; we found that there are some proximity groups between *G. biloba* and *Populus trichocarpa*, suggesting that they are straight homologous proteins, all of which exist in DC subgroups, such as GbDC1-PtDC1A and GbDC2-PtDC1B. As in most species, a typical functional YTH domain exists in each GbYTH protein. Previous studies have demonstrated that most of the aromatic cages responsible for binding and recognizing m^6^A sites in the YTH domain are composed of tryptophan residues (WWW) [35]. In our study, we performed multiple sequence alignment on the protein sequences of the *G. biloba YTH* gene family and found that all members of the *YTH* gene family in *G. biloba* possess aromatic cages composed of tryptophan residues (WWW), which is consistent with previous research findings [35,36]. Subsequently, to help us understand the functions of GbYTH proteins, we conducted subcellular localization experiments for the representative *GbDC1* and *GbECT2*, the homologs of *CPSF30-L* and *ECT2* that have been well studied in *Arabidopsis thaliana*. The experimental results revealed that *GbDC1* was localized in the nucleus and *GbECT2* was localized in both the nucleus and the cytoplasm, aligning with the prediction results.

Studies have shown that m^6^A recognition proteins can directly interact in the cytoplasm and exhibit redundant functions in regulating ABA responses during seed germination and post-germination growth in plants [37]. The cis-regulatory are involved in the regulation of downstream gene expression and intimately related to plant growth and development as well as plant responses to abiotic stresses. Therefore, the cis-regulatory elements in the promoters of *GbYTH* genes were analyzed to verify their functions in response to stresses. The results showed that these *GbYTH* genes play important roles in coping with abiotic stresses. We selected three of these abiotic stresses that are representative, ABA, MeJA, and NaCl. Therefore, we subjected *G. biloba* seedlings to ABA, MeJA, and NaCl stresses and investigated the expression patterns of the *G. biloba YTH* genes after short-term treatments. A substantial number of genes exhibited a decrease in expression, followed by an increase after ABA treatment, indicating that, although ABA stimulation inhibited gene expression in the short term, most genes have a certain resistance to ABA stimulation. Similarly, most *GbYTH* genes also eventually showed an increase in expression after MeJA treatment as well as NaCl stress. Since ABA and MeJA play important roles in plant adaptation to abiotic stresses, such as drought and freezing, and the present study showed that most of the *GbYTH* genes indeed respond to ABA, MeJA, and NaCl stresses, we hypothesized that the *G. biloba YTH* gene family has a significant role in resistance to abiotic stresses. In many plants, genes containing the YTH domain can also respond to various abiotic stresses, including high salinity, drought, heat, cold, and polyethylene glycol stresses. For example, in dicot species Arabidopsis thaliana, the YTHDFA subfamily members ECT2/3/4 are important for nitrate transport [26]. *MhYTP1* and *MhYTP2* participate in MeJA, SA, and ABA signaling, and overexpression of *MhYTP1* or *MhYTP2* makes plants more sensitive to NaCl [38]. *CsYTHs* expression levels were altered under various stresses such as salt, drought, cold, and abscisic acid (ABA) treatments [39]. Similar expression patterns were also observed in tomatoes [30] and other species [31]. In addition, diverse RNAs and RNA-binding proteins form phase-separated, membraneless granules in cells under stress conditions. The m^6^A-modified mRNAs are enriched in stress granule (SGs), and YTHDF proteins that bind to m^6^A are essential for SG formation. Depletion of YTHDF1/3 inhibits SG formation and recruitment of mRNAs to SGs [39]. We therefore hypothesized that the expression of YTH genes in response to hormone stress (ABA and MeJA) and salt stress (NaCl) might be conserved. Furthermore, we analyzed the expression of *G. biloba YTH* gene family in roots, stems, and leaves. The results showed that the expression of different *G. biloba YTH* genes varied greatly among different tissues of *G. biloba YTH*, with higher expression observed in leaves compared to other tissues. Notably, the expressions of *GbDF4*, *GbDF5*, and *GbDC1* were low in all tissues, whereas *GbDF1* had high expression in all tissues, suggesting that this gene might play an important role in the growth and development of *G. biloba*.

In the general study of proteins, it is widely believed that the structure of a protein determines its function. Proteins with a specific disordered domain have been discovered possessing the capacity to easily form droplet-like induced phase transitions. Studies have demonstrated that, in mammals, the cytoplasmic m^6^A-binding proteins YTHDF1, YTHDF2, and YTHDF3 are capable of forming liquid-droplet phase separation both in vitro and in vivo, and this phenomenon is significantly enhanced by mRNAs containing m^6^A residues [28]. Therefore, we performed a liquid–liquid separation prediction of the *G. biloba* YTH proteins, and the results showed the presence of one to four prion-like disordered regions in the predicted sequences of the GbDF1, GbDF2, GbDF3, GbDF4, GbDF6, GbDF7, GbDF8, and GbDC1 proteins, respectively. Prion-like disordered regions are precursors of proteins that have the ability to undergo phase transition, and this study found that the m^6^A-binding activity of CPSF30-L enhances the formation of liquid-like nuclear bodies, where CPSF30-L mainly recognizes m^6^A-modified far-upstream elements to control polyadenylation site choice [40]. Furthermore, this study has shown that m^6^A mRNA modification and its reader protein ECT8 act together as a key checkpoint for negative feedback regulation of abscisic acid (ABA) signaling by sequestering the m^6^A-modified ABA receptor gene PYL7 via phase-separated ECT8 condensates in stress granules in response to ABA [41], thus leading us to hypothesize that these YTH proteins are highly likely to undergo phase segregation in *G. biloba* to exercise certain functions regarding m^6^A.

In this study, a total of 10 *G. biloba YTH* genes were identified and subjected to a comprehensive analysis of their physicochemical properties, phylogenetic analysis, cis-regulatory elements, liquid–liquid phase separation prediction, and expression patterns. Our findings indicate that the *YTH* gene family plays an important role in the growth and development of *G. biloba*, as well as in response to abiotic stresses. Above all, the identification and analysis of the members of the *G. biloba YTH* gene family presented in this study provide a solid theoretical foundation for future research focusing on this gene family.

## Figures and Tables

**Table 1:** Analysis of physical and chemical properties of *G. biloba YTH* gene family.

Name	Gene Name	Subgroup	CDS Length (bp)	Peptide Residue (aa)	Genome Location	PI	Mw (kDa)	Predicted Location (s)
*GbDF1*	Gb_17619	DFA	2121	706	Chr07	6.79	77.267	Nucleus
*GbDF2*	Gb_38284	DFB	1740	579	Chr08	6.97	63.020	Nucleus or Chloroplast
*GbDF3*	Gb_06865	DFB	2130	709	Chr01	5.21	77.236	Nucleus
*GbDF4*	Gb_34757	DFC	1926	641	Chr09	5.80	70.187	Nucleus
*GbDF5*	Gb_16810	DFC	1539	512	Chr06	8.20	57.641	Nucleus
*GbDF6*	Gb_40865	DFC	2340	779	Chr03	5.51	85.087	Nucleus
*GbDF7*	Gb_19406	DFC	1896	631	Chr01	6.23	69.909	Nucleus
*GbDF8*	Gb_40340	DFC	2178	725	Chr07	5.05	79.475	Nucleus
*GbDC1*	Gb_34616	DCA	2274	757	Chr10	6.47	83.098	Nucleus
*GbDC2*	Gb_31165	DCB	1182	393	Chr04	9.24	44.173	Nucleus

## Data Availability

Data are contained within the article.

## Supplementary Materials

The following supporting information can be downloaded at: https://www.mdpi.com/article/10.3390/plants13243589/s1, Table S1: Protein sequence of GbYTHs; Table S2: Amino acid sequence of YTH domain for evolutionary tree construction; Table S3: List of the motifs of GbYTH proteins; Table S4: Prediction of promoter cis-regulatory elements; Table S5: Primer sequences for gene cloning PCR; Table S6: Primer sequences for real-time PCR.
