# Supplementary material for: Genome-Wide Identification and Expression Analysis of Members in the YT521-B Homology Domain-Containing RNA Binding Protein Family in Ginkgo biloba
Source: Plants (Basel). 2024 Dec 23;13(24):3589. doi: 10.3390/plants13243589 (PMC11679001; doi:10.3390/plants13243589)
Supplement: Supplementary file 1 [file plants-13-03589-s001.zip › plants-3311879-supplementary.pdf]

Table S1: Protein sequence of GbYTHs.

Gene Nam	Locus ID	Protein sequence
GbDF1	Gb_17619	MLQMQHNSLLHHNMVLVKLQMALLMHLEQDLQPIELALLSCKTW
GbDF2	Gb_38284	MPYGPYSPAGTPLPTIRADGQLFGTQQYPYPAPFYQQPVPPGVQYI
GbDF3	Gb_06865	MAAVVPPGEETAELLKKLSIDPQQKPAEASEGTHQPQAIQDGVGD
GbDF4	Gb_34757	MASTADTVNQICEKVEALEINPRPSSVGQEKKSSLEAKQAVKCKD
GbDF5	Gb_16810	MHMEGYDLYSDGYQQAALFNGAQYHALGSHNEVTPVFYQPMDA
GbDF6	Gb_40865	MAVAVQPTKQVEELLKSMEIGAESKSTYACEMPAIDRNNSVASGS
GbDF7	Gb_19406	MAVAVPPKQEITELMHNLKLDDQPKSNDVRKIPAKDGNTFEAKA
GbDF8	Gb_40340	MVVAAELEGLNRGFYCGDFWCNPMESEPVAAERQPEKQAEEML
GbDC1	Gb_34616	MDDVEGALSFDFEGGLETGATAGGPSNLGPEATSNVQTPAGPLTT
GbDC2	Gb_31165	MVFPAVCSSMDFGLMGCAYVSQRCPLTSASLGSVSLLPSKSFCTL

LWIQICFMCQTVMPTRPIIMEAYDGRIGEWDDYPQYVNADGVEIPATGVYGDNGSLLY
SSPTPLSQGELSSSVGVDQATFAVDGSNANFVNGNGMTVAPRPGYPMAYGSYGRGVLP
SEGTDTTSPGPSTDEISTPGAQDYVVDPSLLYAPNGYAQQAYYYGCYDGAIGEWEEYP
RTELNHSSPSREDKETTTRGLPSASQNVAGIAGISVAFASYIAPNSHPATQVYSYGGNDA
GCSQAVYSSTRTPIPAFSTINYFYGAQLMHPDCTYLQAASPECKYWSSAVDVPAISRFEC
VLPSSEDTNTMDNEGEQDLLVEQGLYYPASNYYGYYCTGYEPPGVEWDANGRFVGVD
VLPSSDAAAAKENKEEVDTMVDQGLYYPPNNYYGVYYPGHETHGKEWDDHFVGVDA
NLKIDSTAKPPSVDASEAPAKEGSSSDATSSVTSSLGDATSTKESEVEQEDMVEQGAYY
PNANVGQKVSKRNFRQTVCRHWLRGLCMKGEFCGYLHQYDKARMPICRFFARFGEC
CYLKEPKCYFFCFHFNDSTLQLWAHGSSSNKECEKQHYRNNQGRVKCLFNGVVNAGS

HGFGYAPQVPYGPYSPAGTPVPTMGADGQLYGPQPYQYTGPFYQQPVPPGAQYVPSPT
VTITSPGYQDPRHGYDGIRTGGSWSDSAKVSEGQQRPNSGSAVPAVTSQTIPALGPFGQN
GYVNVDGMEIPSPGVYGDSGSVMFHPGYGYPPQISYGSYSPAGTPVPSVRADGQLYGPQ
AFALDSDGYPPSAFVNDFHYHFPGYPNEIAPLFYPPVDGGYSPQTLYSSTGTLIPAFGTDNI
PGTQKNDYLSSFPSGENYFSVGRPALQPYVMVADGPYSRGIFPTTNPAPVNAVVGNCMR
GQEIQYPGLHPESVPLVYYAPGYGYPQPAFNPYNPYIPGAVLGVDGQYIGHQPYYTGPMY
REMQYPGIRAENGSLVYYTSGYGYPQHAYNPYNPYIPGALVGADGQFLGHQPYYTGPA
PANNYYGFYYPGYEAPVSEWDEHGCFIGMDGVEFQYPGIQAENGSLVYYMPSYGYQQP
REQDCVYKHTHEDIKECNMYKLGFCPNGPDCRYRHQKLPGPPPPMEEIFEKIQQQNPTLN
SGAGRLHTRRYFIIKSLSHHNIQMSIEKGIWATQVMNEPVLEEAFQTSERVILIFSVNMSGY

PPLPQGDVSTSVPADQGTLSGDSSNGHTTNGNGNGVTHGPRPGYPMALLPSNGSYGRGI

IRSVSHVTGAQRPPSGGMGPAPGTFNRVYSPNRMFPQGNSSGRGSPGYSAGRSWIAVDK

PFQYPGPFYQQPVSPSTQYIPSPTSVSPGDVSNSAAVNQGSTDGYNGNVTVNGNSPRSGY

FYGHHFQLPGSVYQQAASPECQLWSFPMGLPPGEVVMPGSEDLGIQGSYFHSFQGGSNP

SASTYWMEPTRRVKGHQKNSLSLRTSATSGNTLPTVIFPSPQPQQAVYSPHVTSDIRPPT

YQHPVSSPGYYPPSLQSGSEALPTSSSESGLLVADAVTTSGINVSTTATMPRPGHSAAFMP

YQHPVSSGYFPPVPPPTRKPGSMVADRANANGTSGSSATLKPAEQAGLKGTAHSSGPPS

AYNPYNPYIPGALIGVDGQFLGHQPYYPGAIYQQPVSSPGYFQPPVQYGSEVMPAFAWE

GSYSSRHPQQRHYNHNKDEGSQRKSSMVGPSQRPHGTSSLGEDSSSQQQQQAKHLPQQ

FQGYAQMMSPVGWRRANVWTESNSGANPWGRTFKVKWLRLYNLPFQRTIHLKNPLN

LPANVQNAGYHDGIRSVGPWLDGPVASEGQLRPATSTPVSSVVTQTTSPGPLGQNIRPLP
NKQRGRGNGSLCNCIGPLDVLNEQNRGPRTARFRNQQMLPGSARLVKEQSLTMNGNNE
PLALLPSHAPYGRVVSPVPIPSLGYQDPRFGYDGGRISGPWADSSKLSEGHQSPASTNGY
FFGGRPGFQPYLMTPNGSYGSGILPAALTGTTPVNAAIGYDSMRPAIAHWMDPRNVGEH
GKYFGISSPGKQRSIINGVTSNQSETVKNNWGWSLENNGQLVNEASLGCEQGMQGGLND
THVPFSRDMQSVGMATSSAQESKSLARSGEGQRRPAVSAGQQIPQGIMTSTSLPYQSFRH
QSFRPMSQIQLNQQQGSGFRGAVLAKDYIPFRKFAPGTIQGKGNVPFTNSSVGFRPNGRG
SGLPVADRANGNGFSGSPTTGGPRQGYSVGTIPTHVPYLKSVPPAGKQSTAASDSKVSAQ
HASQIQVQSMLQNPIGNGLSSQPNTVSAASPLPQGHSRYFIVKSSNLENLELSVQRGMWA
EYKPVKISRDCQELTQEIGEALCSLIDGGAELDGKQKRKMTVRDAALTKRLHRDSPSAMA

HGVQHPRPPSGMAPAPGYMNRIYPSNRTYPPNNYSGRSGPNYGANGLDSRPNGRSWIA
ISNVAVNRELYNQPEFVTKYTDAKFFIIKSYSEDNIHKSIKYGVWASTQNGNKKLDAAY
TTGSQVPTQMVPSPGHPVQNVHPHMMHNVQRAQQQWSPSGNGTGPGPMNRFYSPNRQ
QKFSVSSGVHAAVGESVPSVGLRPLQPQQLVYSPHVTAGLHPLSVDSFEIPLPGTQWRM
EERSRTARSRTQWLTINRQEREKSEKLLQVDEILSFLMENDCFNRSDFVTKYDDAKFFVI
IMNQNLQLNQQQGSSIQGPVQAKGYLPFGKVPPSANQGKATLPLINNFTDLKLNERGYV
RGKVNGNLSNENWNLDVLTEQNRGPRTNRIRNHPIASSVLKDAGNQAVVGNSEVYGLA
TSEGPQAPPVSVAQITTQGNMPSTGLRTQCLRPLSKIPQQVSTIQAAVLPKGYLPLGKVT
THRNNEGKLNEAFDSCDNVILVFSVNGTRHFQGCARMTSKIGGVVGGAGWKYANGTS
ADGKYLGGIPISQLSWPMTPMLYSTTQYPAGTPHEPYDPSGGLGPKRISSHLEEPKSSRLR

VDKYRPRGKGNGSMTNSNESLDILNEQNRGPRTARFRNQRMVPGAAWPVKGQSSTNGS
REAQEKTGSCPIFLFFSVNASGQFCGVAEMVGPVDFNKSVDYWQQDKWSGQFPVKWHII
QYPQNNNSGRFSPGYGIGSFDSRGNGRSWIAIDKSKPRGRGIMFNGNDNLDVLNEQNRGP
NDGLISYPMQTGGNNSGLVVDNKRLMGSRALLDNGQGSLGSSNDSVHHQTVRSVNHQM
KSYSEDDIHKSIKYNLWASTPIGNLKLNQAYKDAQKRAGDKPGGCPVFFFFSVNGSGRFC
GADRLKQRGQINVNLTNGNGNLDVLSEQNRGPRTNRTRNQWISPAVHNAGTDEVVGNV
TNRDQYNRADFSTTYDAAKFFVIKSYSEDDVHKSIKYNVWASTPNGNKRLDEAYQDAQ
ACSNQGKGTVLYSNDTAEFKPNGRGWVGGDRLKMRGKVNGTGNLDALNEQNRGPRIN
HYGRNFRLKWLKLCELSFHKTHHLRNPFNENLPVKISRDCQELELSIGEQLVSLLYLEPDS

SEDANVVPNRDQYNRPEFVTKYSDAKFFIIKSYSEDDIHKSIKYSVWASTPNGNKKLDAA
KDVSNSQLRHITLENNDNKPVTNSRDTQEVKFEQGIEMLNIFKNYVSKMSILDDFQFYES
RTARFRNQQGSSGPLQPGRGQIPTSNGNGEENNVVPNREQYNRPEFVTSYSDAKFFIIKS
TSSSRQANGEVAFPDRVNEILSVLGDGDSYNRPDFVTNYDDAKFFVIKSYSEDDIHKSIK
GLAEMVGPVDFTKSMDFWQQGKWNGSFSVRWHIIKDIHNSQLRHILLKNNDNKPVTNS
EGDNPAGHIVQYNRDDFDINCDDARFFVIKSYSEDDVHKSIKYSVWSSTPNGNKRLDGA
QTSGVRSGSCPVFLFFSVNASGQFCGVAEMIGSVDFNKSMDFWQQDKWSGFFPVKWHM
RMRNPWLSPVDVSPGSQGVVGNTEGYSPAVNTDQYNLADFSTKFDNALFFVIKSYSEDD
ELMAVACAVESKREEEKAQGVNSAHESEDPNIVPFEDNDDDQDEDESDEEESSSQTRSA

YKESQEKSGGCPIFLFFSVNASGQFCGVAEMVGPVDFDQSVDYWQQDKWSGRFLVKW
RQKAMQEKRARQQASYQSGGEFEPDDPLKAQEGNAGEAAAEVDIKLRQTPADLTSALP
SEDDIHKSIKYNVWASTPNGNKKLDAAYKEAHEKNGGCPVFLFFSVNASGQFCGVAE
NVWASTPNGNLKLNQAYQDAQRRAGDNPGGCPIFFFFSVNGSGRFCGIAEMVGPVDFS
RDAQEVSLAEGLEMLNIFKNCSSRSSILDNFYLYESRQRALQDKRDREKAQLQ
YQDAQERSGGKPGGCPVFLFFSVNASGQFCGVAEMVGPVNFKSNKDFWQQDKWSGCF
IKDIPNSQFRQIILENNDNKPVTNSRDTQEIKYGQGVEMLNIFKNYSFKTSILDDFMFYEG
VHKSIKYNVWASTPNGNKRLDVAYQAAQDRSGGKPGSCPFFSEFSVPRTPATYDTFDF
QGRGRGRGTMWRGPMARVGRGIAGSKVVGFHPGMVPGDGYGFDRFGMGPADGFAM

HIVKDVPNSQLRHITLENNDNKPVTNSRDTQEVKYDQGIEMLKIFKDYVSKTSILDDFVF
PSDGLSCEGGWNKNNPSQKLSLEERGDLSSVGDALKAAKPTTDEGDSIQDEEADKKVP
IVGPVDFIKSVDYWQQDKWSGSFPVKWLIIKDVPNGQFRHITLENNDNKPVTNSRDTQE
KSMDFWQQDKWAGSFSVRWHIIKDIPNSQLRHIILESNDNKPVTNSRDTQEINIQEGLEM

PVKWHIIKDVSNSHLRHIILENNENKPVTNSRDTQEVKYAQGMEMLNIFKNYSWKTSILD
RQKAMKDKKLRQQGQQQQELAACIGANIEQNNLEEQFSYKKGEEEEVPKSCDLKVAEL
KTMNFWQQDKWSGFFPVKWHIIKDIPNSQFRHIILENNENKPVTNSRDTQEFCSIAFGLD
PDMFTAPAGQGRGFPPYGQPGPRYGPASGMMFAPMDGSGPTPGIVFPARPPPPNGIFSH

YEGRQKAMQEKRARQQAQQQQQKQGTFRPGSEFDQLHTEDSVPKACDGKTTGAVYED

VKFEQGTEMLNIFKNYLCKTSILDDFLYYEGHQKTMQEKKARQQAQQQQQQQQVSHA
LNIFKNYPARSSIVDDFYLYESRQRALQEKRARQNAQLHAHQVANLSMEHFPDGLHNK

DFDYYEDREKAMQDKKSRQHAKQQQQLQPAARVGPHIQSKKSDEEFSKLDGEVGRPS
SSLLPSANGSQLSALNDINSVVVDHSLERKEEMAQSKGIMSKEIISKEANTESQIPVSEN
VGSVAKMSMLFFEIKFAQGIEMLNIFKNFSLKTSILDDFMFYESRQKAMQDKKTRQPTQ
APGMMPSGASSHPPMLGSTSPYVPMGAGRSNFMTGPGAVGRPNRPKGMPYRPPQGGN

GKSRLQKPADLTSGATSASGDQYHEGGQSKPDQSSPSEERAVPTVVGEAPKGAKSNLGK

AGVESEVAENLQESISSDAAVENKDGIGSLGSADQSYKNVLTNQTQIPAFNERKESSMVA

DLKASDRSLPLSCVNGGPRSESFSTKQTISGSIVRMKDETVSLKVVDSKKDDPKGVSCDGI

QQQFQAAARVDVQQKVLDEQLCKLDEYKVSKPSDLSSPMSSGNGNQPSDSRKSVPSGSE
SGRGRRDQKRRRADHNNSNDRLLSGLDQRATGKPWLRQPSGEGLEADREPEFHGPSRL

ESVLTVGTMAIDSRGVNYDSPCVLTVGTMAIDTRVCDPRILTVGTMPIDTNAVDSDFPGG

QHQDGYGYGNNSYPVNNDEDSESEDEAPRRSRHGESKKRRREWDGEEAADQADHCEL[illegible]

LRVGTVFIDQNKGNRDSQSISTVGTVSV
